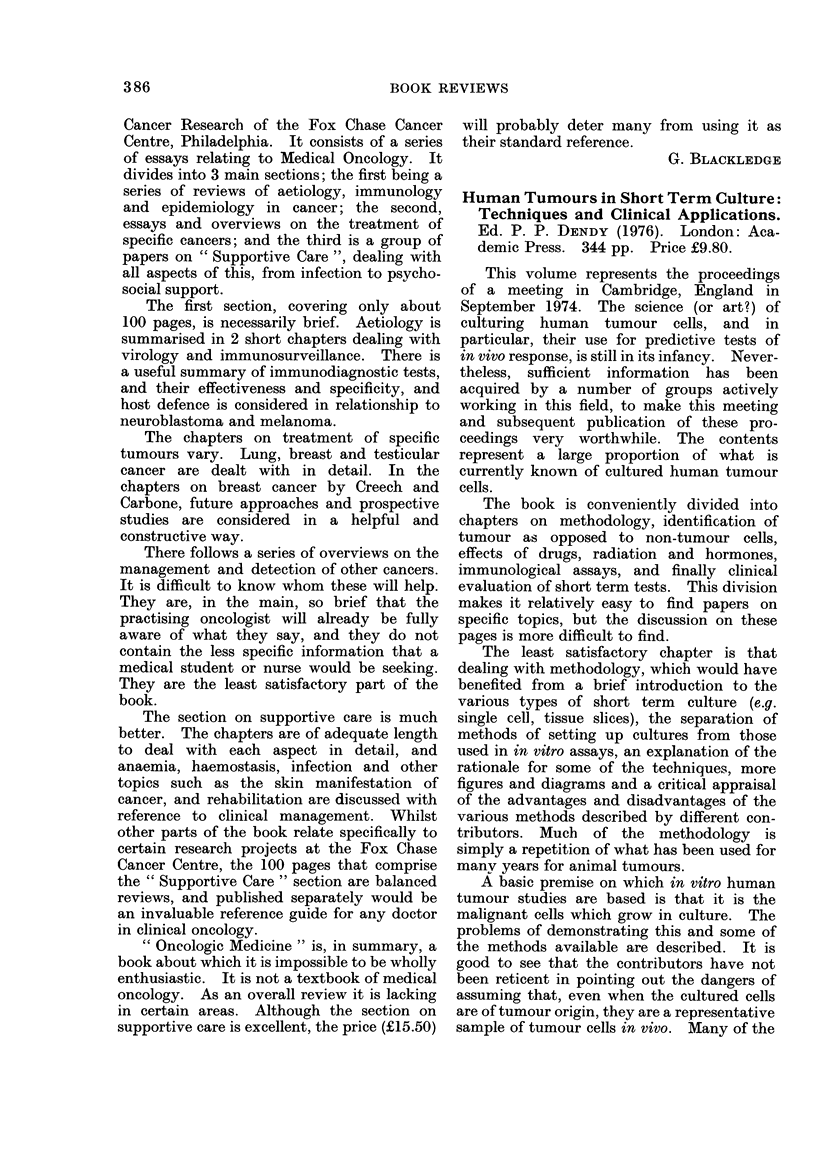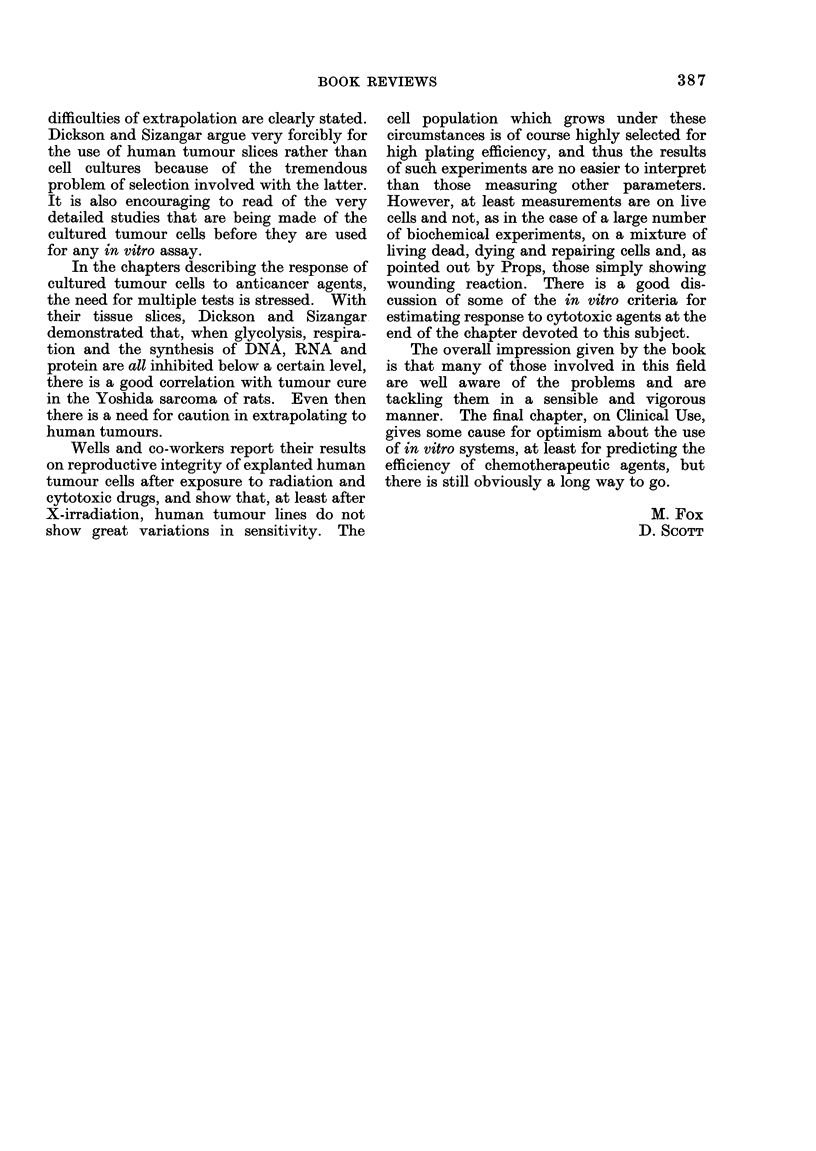# Human Tumours in Short Term Culture: Techniques and Clinical Applications

**Published:** 1977-03

**Authors:** M. Fox, D. Scott


**Human Tumours in Short Term Culture: Techniques and Clinical Applications.** Ed. P. P. Dendy (1976). London: Academic Press. 344 pp. Price £9.80.

This volume represents the proceedings of a meeting in Cambridge, England in September 1974. The science (or art?) of culturing human tumour cells, and in particular, their use for predictive tests of *in vivo* response, is still in its infancy. Nevertheless, sufficient information has been acquired by a number of groups actively working in this field, to make this meeting and subsequent publication of these proceedings very worthwhile. The contents represent a large proportion of what is currently known of cultured human tumour cells.

The book is conveniently divided into chapters on methodology, identification of tumour as opposed to non-tumour cells, effects of drugs, radiation and hormones, immunological assays, and finally clinical evaluation of short term tests. This division makes it relatively easy to find papers on specific topics, but the discussion on these pages is more difficult to find.

The least satisfactory chapter is that dealing with methodology, which would have benefited from a brief introduction to the various types of short term culture (*e.g.* single cell, tissue slices), the separation of methods of setting up cultures from those used in *in vitro* assays, an explanation of the rationale for some of the techniques, more figures and diagrams and a critical appraisal of the advantages and disadvantages of the various methods described by different contributors. Much of the methodology is simply a repetition of what has been used for many years for animal tumours.

A basic premise on which *in vitro* human tumour studies are based is that it is the malignant cells which grow in culture. The problems of demonstrating this and some of the methods available are described. It is good to see that the contributors have not been reticent in pointing out the dangers of assuming that, even when the cultured cells are of tumour origin, they are a representative sample of tumour cells *in vivo*. Many of the difficulties of extrapolation are clearly stated. Dickson and Sizangar argue very forcibly for the use of human tumour slices rather than cell cultures because of the tremendous problem of selection involved with the latter. It is also encouraging to read of the very detailed studies that are being made of the cultured tumour cells before they are used for any *in vitro* assay.

In the chapters describing the response of cultured tumour cells to anticancer agents, the need for multiple tests is stressed. With their tissue slices, Dickson and Sizangar demonstrated that, when glycolysis, respiration and the synthesis of DNA, RNA and protein are *all* inhibited below a certain level, there is a good correlation with tumour cure in the Yoshida sarcoma of rats. Even then there is a need for caution in extrapolating to human tumours.

Wells and co-workers report their results on reproductive integrity of explanted human tumour cells after exposure to radiation and cytotoxic drugs, and show that, at least after X-irradiation, human tumour lines do not show great variations in sensitivity. The cell population which grows under these circumstances is of course highly selected for high plating efficiency, and thus the results of such experiments are no easier to interpret than those measuring other parameters. However, at least measurements are on live cells and not, as in the case of a large number of biochemical experiments, on a mixture of living dead, dying and repairing cells and, as pointed out by Props, those simply showing wounding reaction. There is a good discussion of some of the *in vitro* criteria for estimating response to cytotoxic agents at the end of the chapter devoted to this subject.

The overall impression given by the book is that many of those involved in this field are well aware of the problems and are tackling them in a sensible and vigorous manner. The final chapter, on Clinical Use, gives some cause for optimism about the use of *in vitro* systems, at least for predicting the efficiency of chemotherapeutic agents, but there is still obviously a long way to go.

M. Fox
D. Scott